# Asenapine for delirium in patients with cancer: A scoping review

**DOI:** 10.1002/pcn5.70307

**Published:** 2026-02-26

**Authors:** Tetsuro Ishida, Makoto Kobayakawa, Jun Kako, Yoshinobu Matsuda, Ryoichi Sadahiro, Hitoshi Tanimukai

**Affiliations:** ^1^ Delirium Subcommittee, Guideline Development Committee Japan Psycho‐Oncology Society Tokyo Japan; ^2^ Department of Psychiatry Hiroshima Red Cross Hospital & Atomic‐Bomb Survivors Hospital Hiroshima Japan; ^3^ Graduate School of Medicine Mie University Tsu Japan; ^4^ Department of Psychosomatic Internal Medicine NHO Kinki Chuo Chest Medical Center Sakai Japan; ^5^ Department of Psycho‐Oncology, National Cancer Center Hospital National Cancer Center Japan Tokyo Japan; ^6^ Graduate School of Nursing Nagoya City University Nagoya Japan

**Keywords:** antipsychotic agents, asenapine, delirium, neoplasms, palliative care

## Abstract

This scoping review mapped the existing literature on the application of asenapine for delirium management in patients with cancer, including prevention and treatment, while summarizing current research trends. The review followed the methodological framework proposed by Arksey and O'Malley and was reported in accordance with the Preferred Reporting Items for Systematic reviews and Meta‐Analyses Extension for Scoping Reviews. PubMed and Ichushi‐Web were searched on June 30, 2025, using the keywords “asenapine,” “delirium,” and “cancer.” Eligible studies included reports on asenapine management for delirium in patients with cancer. Two reviewers independently screened records and extracted data. Among the six records identified, three studies met the inclusion criteria and were included in this review. Two of these were retrospective observational studies, and one was a case report. One retrospective study described six terminally ill patients treated with sublingual asenapine, showing marked improvement in agitation, as measured using the Richmond Agitation‐Sedation Scale. The other study analyzed 20 patients with advanced cancer and reported reductions in the Agitation Distress Scale scores, particularly among those with poor performance status. Moreover, this case report highlighted the successful use of sublingual asenapine in a patient with aphagia unresponsive to other antipsychotic agents. Across the studies, asenapine was generally well‐tolerated, with one suspected dysarthria and no serious adverse events. The current evidence is limited in quantity and quality, and no preventive studies were identified. While preliminary findings suggest that sublingual asenapine may be useful and well‐tolerated for treating delirium in patients with cancer, further high‐quality studies are warranted.

## INTRODUCTION

Delirium is a neurocognitive syndrome characterized by acute disturbances in attention, awareness, and cognition.^1^, ^2^ It is highly prevalent in patients with advanced cancer, occurring in approximately 30%–40% of individuals at admission to palliative care units and in up to 90% in the last days of life.^3^, ^4^ In this setting, factors such as the inability to take oral medications and difficulties in maintaining intravenous access usually make pharmacological treatment challenging to administer. Delirium is associated with considerable distress for patients, families, and healthcare providers, and its management remains a major clinical challenge.^5^

Non‐pharmacological interventions, such as reorientation, environmental modification, sleep hygiene, and sensory support, are primarily recommended as the first‐line approach for preventing delirium in patients with cancer.^6^ However, pharmacological treatment may be a useful therapeutic option when patients present with agitation or hyperactive symptoms.^7^ In this context, antipsychotics are only conditionally recommended in clinical guidelines, and their use is generally restricted to cases with severe agitation or distress that cannot otherwise be managed.^7^, ^8^, ^9^ Antipsychotics used in these cases have long been dominated by haloperidol, risperidone, and olanzapine, but recently, asenapine has come into use.

Asenapine is a second‐generation antipsychotic classified as a multi‐acting receptor‐targeted antipsychotic.^10^ A practical advantage is its sublingual formulation, which allows administration without water, bypasses the gastrointestinal tract and most first‐pass metabolism, and provides a relatively rapid onset of effect. Pharmacokinetic studies also indicate that systemic exposure (overall blood concentration) remains relatively stable in patients with mild to moderate hepatic impairment (although contraindicated in severe hepatic dysfunction).^11^ These features are particularly useful in patients with impaired swallowing, difficulty maintaining intravenous access, or severe nausea and gastric discomfort, even without obstruction. Moreover, sublingual administration avoids the need for additional fluid intake, which may be advantageous in patients requiring strict fluid restriction (e.g., hyponatremia management) or in those at high risk of aspiration. Pharmacologically, asenapine predominantly acts as an antagonist (e.g., dopamine D2/D3/D4 and serotonin 5‐HT2A/5‐HT2C),^12^ with relatively weaker muscarinic (M1) binding than olanzapine.^13^ Consequently, it may impose a lower anticholinergic burden. These properties suggest that asenapine is a useful therapeutic option for delirium in patients with cancer, particularly in advanced stages.

However, evidence for the use of asenapine in cancer‐related delirium has not been systematically reviewed. Therefore, this scoping review aimed to comprehensively map the existing literature on the use of asenapine for delirium in patients with cancer—within the overall context of delirium management, encompassing both prevention and treatment—and summarize research trends and reported findings.

## METHODS

### Study design

This scoping review was conducted to comprehensively explore and map the existing literature on the use of asenapine for treating delirium in patients with cancer. The methodological framework proposed by Arksey and O'Malley^14^ was adopted, and reporting followed the Preferred Reporting Items for Systematic reviews and Meta‐Analyses (PRISMA) extension for Scoping Reviews.^14^, ^15^ The study protocol was prospectively registered with the University Hospital Medical Information Network (UMIN000058326).

### Research question

The guiding research question was: What evidence exists regarding the use of asenapine for delirium management in patients with cancer?

### Inclusion and exclusion criteria

Studies were included if they involved adult patients aged ≥18 years who had cancer and delirium, and if asenapine was administered for delirium management. Eligible outcomes included any clinical parameters related to delirium, such as agitation, sedation level, medication use, overall clinical course, and adverse events or safety profiles. Primary research articles, including randomized controlled trials (RCTs), non‐randomized studies, observational studies, and case reports, were considered.

Studies were excluded if they were duplicate publications, conference abstracts without full data, narrative reviews or commentaries, or study protocols without results. Studies primarily targeting other psychiatric disorders (e.g., schizophrenia and bipolar disorder) or neurological diseases (e.g., epilepsy, Parkinson's disease) and articles not written in English or Japanese were also excluded.

### Information sources and search strategy

PubMed and Ichushi‐Web (Japan Medical Abstracts Society) were systematically searched. The initial search was conducted on June 30, 2025. PubMed was selected to capture international literature, while Ichushi‐Web was included to identify studies published in Japanese, since most reports on asenapine use for treating delirium in patients with cancer originated from Japan. Other databases, such as Embase or Cumulative Index to Nursing and Allied Health Literature, were not searched because preliminary scoping indicated that relevant studies were unlikely to be indexed outside PubMed and Ichushi‐Web.

The search strategy combined controlled vocabulary (Medical Subject Headings terms) and free‐text terms related to “asenapine,” “delirium,” and “cancer.” For example, the terms “asenapine,” “delirium,” and “neoplasms/cancer” were combined using Boolean operators in PubMed. The full search strategies for each database are provided in Appendix 1.

### Study selection

**Figure 1:**
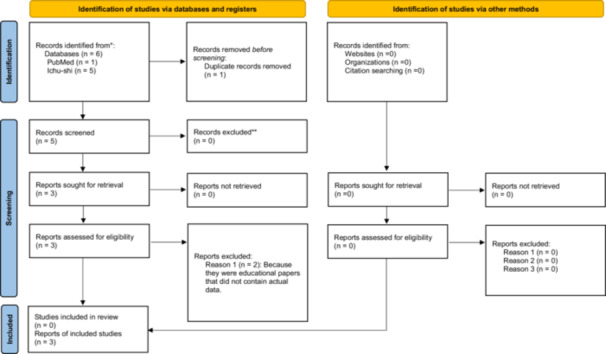
Preferred Reporting Items for Systematic reviews and Meta‐Analyses (PRISMA) 2020 flow diagram for new systematic reviews, which included searches of databases, registers, and other sources. *Consider, if feasible to do so, reporting the number of records identified from each database or register searched (rather than the total number across all databases/registers). **If automation tools were used, indicate how many records were excluded by a human and how many were excluded by automation tools. *From:* Page et al.^16^ For more information, visit: http://www.prisma-statement.org/.

All identified records were exported, and duplicate entries were removed prior to screening. Two reviewers independently screened titles and abstracts to exclude irrelevant reports. For potentially relevant records, full texts were retrieved and comprehensively assessed. Disagreements regarding eligibility were resolved through discussion, and a third reviewer was consulted if consensus could not be reached (Figure 1).

### Data charting

A standardized data extraction form was developed to collect key study characteristics, including the first author, year of publication, and country; study design and setting; patient population and sample size; details of the intervention such as asenapine dose, route, and duration; comparator or prior treatment if applicable; outcomes and assessment tools (e.g., Richmond Agitation‐Sedation Scale [RASS],^17^ Agitation Distress Scale [ADS],^18^ and Memorial Delirium Assessment Scale^18^); and main findings, including reported adverse events. Two reviewers independently extracted the data, with discrepancies resolved through consensus.

### Synthesis

The included studies were collated and narratively summarized. No formal risk of bias or quality assessment was conducted in accordance with scoping review methodology. Instead, the focus was on mapping the available evidence and identifying research gaps.

## RESULTS

### Study selection

The initial search identified a total of six records (PubMed: *n* = 1; Ichushi‐Web: *n* = 5). Five unique records were screened at the title and abstract level after removing one duplicate. Among these, two Japanese papers were excluded because they were educational articles without original clinical data. Finally, three studies met the inclusion criteria: two retrospective observational studies and one case report, all of which were conducted in Japan. The study selection process is summarized in the PRISMA 2020 flow diagram (Figure 1). Table 1 summarizes the included studies.

**Table 1:** Summary of included studies.

First author (year)	Country	Study design	Sample size/population	Intervention (asenapine dose and route)	Duration	Outcome measures	Main findings	Adverse events
Nakano (2021)	Japan	Retrospective observational	6 terminally ill cancer patients	Sublingual 5 mg, regular or PRN	2–9 days (median 4)	RASS	RASS improved from +2.8 to –1.8 (estimated); no EPS or respiratory suppression	None reported
Maekura (2023)	Japan	Retrospective observational	20 advanced cancer patients	Sublingual 2.5–15 mg (mean 3.9 initial, 5 final)	Mean 10.9 days (range 1–86)	ADS	ADS total score improved 12 → 7.9 (*p* < 0.001); significant effect in PS 3–4; non‐significant in PS 1–2	1 suspected dysarthria
Osawa (2019)	Japan	Case report	79‐year‐old female, recurrent head and neck SCC, aphagia	Sublingual 2.5–10 mg, tapered to 2.5 mg	Several days, then maintenance	Clinical observation	Rapid symptom improvement despite failure of oral haloperidol, risperidone, chlorpromazine, levomepromazine	None reported

Abbreviations: ADS, Agitation Distress Scale; EPS, extrapyramidal symptoms; PRN, pro re nata; RASS, Richmond Agitation‐Sedation Scale; SCC, squamous cell carcinoma.

### Summary of findings

Across the three eligible studies, sublingual asenapine consistently demonstrated clinical effectiveness in reducing agitation and improving delirium symptoms in patients with cancer. In the retrospective study by Nakano et al., terminally ill patients showed improvements within days of initiation, with reductions in RASS scores of approximately 5 points.^19^ Similarly, the study by Maekura et al. provided supportive evidence in a larger cohort, showing statistically significant reductions in ADS scores, particularly among patients with poor performance status (PS 3–4).^20^ The case report by Osawa et al. described a patient with recurrent head and neck squamous cell carcinoma, in whom several oral antipsychotics were ineffective, whereas asenapine led to an improvement in delirium.^21^ All three studies suggested that asenapine was generally well‐tolerated, with only one suspected adverse event reported (dysarthria).

### Safety profile

The overall safety profile of asenapine was favorable, with only one report of suspected dysarthria. No severe adverse events, such as extrapyramidal symptoms or respiratory depression, were reported.^21^

## DISCUSSION

### Key findings

Three studies examining the use of sublingual asenapine for treating delirium in patients with cancer were identified in this scoping review. The existing evidence was limited to two small‐scale retrospective analyses and a single case report. No clinical trials have been conducted to date.

### Efficacy and safety

Existing reports suggest that sublingual asenapine may improve agitation or delirium symptoms in patients with cancer and do not indicate major safety concerns. However, it should be noted that sublingual administration may be associated with specific adverse effects. These may include oral discomfort, dysgeusia, oral numbness, or incomplete dissolution. In patients with cancer and delirium, such factors could potentially limit the feasibility or tolerability of sublingual administration and should be considered in clinical practice and future research. Moreover, the available evidence remains insufficient to draw definitive conclusions regarding efficacy or safety. Although not directly related to asenapine, an RCT of haloperidol and risperidone failed to demonstrate efficacy for delirium and raised safety concerns in terminally ill patients.^10^ These findings highlight the challenges in establishing effective and well‐tolerated pharmacological treatment for delirium. While current clinical evidence on asenapine remains limited, mechanistic insights may provide additional perspectives. Based on its receptor profile, asenapine may offer relatively favorable tolerability compared with other antipsychotics.^12^, ^13^ This potential advantage may be particularly important in frail patients with advanced cancer, in whom adverse events can substantially worsen prognosis and quality of life.

### Practical advantages and evidence gap of sublingual asenapine

Consistent with both international and Japanese guidelines, antipsychotics are conditionally recommended for patients with cancer who experience severe hyperactive delirium accompanied by marked agitation or distress that compromises safety or care delivery.^7^, ^8^, ^9^, ^22^ However, oral administration of antipsychotics is usually difficult in terminally ill patients, and maintaining intravenous access may not be feasible.^9^ In this context, the sublingual formulation of asenapine offers a practical advantage, as it can be administered without water and provides a rapid onset of action, making it a potentially valuable option in carefully selected cases.

From a research perspective, several important questions remain to be addressed. For example, prospective studies comparing sublingual asenapine with other antipsychotic drugs are required. In addition, dose optimization studies are needed to clarify the minimum effective dose and safety profile in frail patients with advanced cancer.

Although prevention was included within the scope of this review, no studies evaluating asenapine for delirium prevention were identified. In general, pharmacological prevention of delirium using antipsychotic drugs has been limited, owing to concerns regarding adverse effects and ethical considerations, particularly in vulnerable populations. Beyond these general concerns, asenapine has formulation‐specific adverse effects, such as oral discomfort, dysgeusia, and oral numbness, as discussed above. Consequently, opportunities for its use as a preventive agent for delirium may be even more limited in clinical practice. Nevertheless, exploratory research examining its potential preventive role in carefully selected high‐risk populations may still be warranted.

### Limitations

This study has some limitations. First, the search was limited to studies published in English and Japanese, reflecting the languages most commonly used in this research field in Japan. While this approach aligns with scoping review methodology, potentially relevant studies in other languages may have been omitted. Second, in accordance with scoping review methodology, we did not conduct a formal risk‐of‐bias or methodological quality assessment. Consequently, our conclusions are not intended to evaluate the strength of evidence regarding the effectiveness or safety of asenapine. Third, the available evidence was limited to small‐sized, retrospective Japanese studies with heterogeneous dosing and co‐medications, thereby constraining precision and generalizability. Fourth, although preliminary searches of Embase, Scopus, and PsycINFO did not identify eligible studies, this limited database coverage represents a potential limitation. Finally, all existing reports on the use of asenapine have been limited to delirium treatment, and no study has investigated its use for prevention. Therefore, the overall role of asenapine in delirium management should be interpreted cautiously.

## CONCLUSION

This scoping is the first summarizing of clinical data on asenapine, and the review identified three studies on the use of sublingual asenapine for treating delirium in patients with cancer. These include two retrospective observational studies and one case report. Moreover, high‐quality research evaluating the use of asenapine for delirium treatment in patients with cancer is warranted.

## AUTHOR CONTRIBUTIONS

All authors contributed to the conception and design of this scoping review. Tetsuro Ishida and Makoto Kobayakawa independently conducted the literature search, study selection, and data extraction. Tetsuro Ishida drafted the initial version of the manuscript. All authors critically reviewed, revised, and approved the final version of the manuscript.

## CONFLICT OF INTEREST STATEMENT

Dr. Ishida has received lecture fees from Otsuka Pharmaceutical Co. Ltd., Shionogi & Co. Ltd., and Sumitomo Pharma Co. Ltd. Dr. Kobayakawa has received lecture fees from Shionogi & Co. Ltd. Dr. Matsuda has received honoraria from Chugai Pharmaceutical Co., Ltd., AstraZeneca Co. Ltd., Daiichi Sankyo Co. Ltd., Eisai Co. Ltd., Sanofi K.K., and Kyowa Kirin Co. Ltd. Dr. Sadahiro received lecture fees from Hisamitsu Pharmaceutical Co. Inc., Fujimoto Pharmaceutical Corporation, Takeda Pharmaceutical Co. Ltd., Nippon Boehringer Ingelheim Co. Ltd., Chugai Pharmaceutical Co. Ltd., AstraZeneca Co. Ltd., Daiichi Sankyo Co. Ltd., and Eisai Co. Ltd. Dr. Tanimukai has received Global Medical Grants from Pfizer Japan Inc. The remaining authors declare no conflicts of interest.

## ETHICS APPROVAL STATEMENT

This is not applicable, as this is a scoping review of previously published studies and did not involve human participants or animal subjects.

## PATIENT CONSENT STATEMENT

This is not applicable since all patient data referenced in this review were previously published in the original articles.

## CLINICAL TRIAL REGISTRATION

The protocol of this scoping review was prospectively registered with the University Hospital Medical Information Network (UMIN000058326).

## Supporting information

Supporting Information.

## Data Availability

Data sharing is not applicable to this article as no datasets were generated or analyzed during the current study.
