# Supplementary material for: Asenapine for delirium in patients with cancer: A scoping review
Source: PCN Rep. 2026 Feb 26;5(1):e70307. doi: 10.1002/pcn5.70307 (PMC12936851; doi:10.1002/pcn5.70307)
Supplement: Supplementary file 1 — Supporting Information. [file PCN5-5-e70307-s001.docx]

**Search keywords and screening results**

[Primary screening]

**PubMed** (Search date: June 30, 2025)

#1·····Asenapine /TH or Asenapine /AL····················· five cases

#2·····Delirium /TH or Delirium /AL ···························5,507 cases

#3 ····Neoplasms /TH or Neoplasms /AL·····················10,567 cases

#4 ····#1 AND #2 AND #3 NOT PT ("Conference Proceedings")············one case

Of the single reference obtained as a result of the search, one was selected for secondary screening based on a review of the titles and abstracts.

**Ichushi Web** (Search date: June 30, 2025)

#1·····Asenapine /TH or Asenapine /AL·····················203 cases

#2·····Delirium /TH or Delirium /AL··························18,221 cases

#3·····Neoplasms /TH or Cancer/AL···························2,632,701 cases

#4·····Neoplasms /TH or Carcinoma/AL·····················2,835,045 cases

#5·····Neoplasms /TH or Neoplasms /AL····················2,740,787 cases

#6 ·····Neoplasms /TH or Malignant tumors /AL··········2,520,406 cases

#7·····#1 AND #2 AND (#3 OR #4 OR #5 OR #6) NOT PT ("Conference Proceedings")···················· five cases

Of the five documents obtained as a result of the search, three were selected for secondary screening based on a review of the title and abstract.

[Secondary screening]

Among the three documents selected in the primary screening, two were selected as a result of a careful reading of the full text.
